# Head to head comparison of 2D vs real time 3D dipyridamole stress echocardiography

**DOI:** 10.1186/1476-7120-6-31

**Published:** 2008-06-20

**Authors:** Silvia Varnero, Patricia Santagata, Lorenza Pratali, Massimiliano Basso, Alfredo Gandolfo, Paolo Bellotti

**Affiliations:** 1 Servizio di Cardiologia, Ospedale San Paolo, Savona, Italy; 2 Institute of Clinical Physiology CNR, Pisa, Italy

## Abstract

Real-time three-dimensional (RT-3D) echocardiography has entered the clinical practice but true incremental value over standard two-dimensional echocardiography (2D) remains uncertain when applied to stress echo. The aim of the present study is to establish the additional value of RT-3D stress echo over standard 2D stress echocardiography. We evaluated 23 consecutive patients (age = 65 ± 10 years, 16 men) referred for dipyridamole stress echocardiography with Sonos 7500 (Philips Medical Systems, Palo, Alto, CA) equipped with a phased – array 1.6–2.5 MHz probe with second harmonic capability for 2D imaging and a 2–4 MHz matrix-phased array transducer producing 60 × 70 volumetric pyramidal data containing the entire left ventricle for RT-3D imaging. In all patients, images were digitally stored in 2D and 3D for baseline and peak stress with a delay between acquisitions of less than 60 seconds. Wall motion analysis was interpreted on-line for 2D and off-line for RT-3D by joint reading of two expert stress ecocardiographist. Segmental image quality was scored from 1 = excellent to 5 = uninterpretable. Interpretable images were obtained in all patients. Acquisition time for 2D images was 67 ± 21 sec vs 40 ± 22 sec for RT-3D (p = 0.5). Wall motion analysis time was 2.8 ± 0.5 min for 2D and 13 ± 7 min for 3D (p = 0.0001). Segmental image quality score was 1.4 ± 0.5 for 2D and 2.6 ± 0.7 for 3D (p = 0.0001). Positive test results was found in 5/23 patients. 2D and RT-3D were in agreement in 3 out of these 5 positive exams. Overall stress result (positive vs negative) concordance was 91% (Kappa = 0.80) between 2D and RT-3D. During dipyridamole stress echocardiography RT-3D imaging is highly feasible and shows a high concordance rate with standard 2D stress echo. 2D images take longer time to acquire and RT-3D is more time-consuming to analyze. At present, there is no clear clinical advantage justifying routine RT-3D stress echocardiography use.

## Background

Two-dimensional dipyridamole stress echocardiography, is an established and validated method for both the diagnosis and prognosis [1-5] of patients with known or suspected coronary artery disease. However grounds for an accurate interpretation in stress echo rest on two important features: first-acoustic windows that permits complete endocardial border visualization within proper planes of left ventricle (LV) and secondly-prompt acquisition of peak images pertaining predictive accuracy. Unfortunately, attempting to image a three-dimensional structure, such as the heart, by a 2D device requires multiple windows and time, with the danger of still producing insufficient tomographic view and losing important information between section planes [6]. Real-time three-dimensional (RT-3D) echocardiography has entered the clinical practice [7] but true incremental value over standard two-dimensional echocardiography (2D) remains uncertain in stress echo. The three-dimensional echocardiography (3D) has the theoretical potential to more completely assess LV [8-12] but previous 3D imaging system were tedious techniques that used off-line reconstruction of multiple 2D images [8-10], not suitable for stress echocardiography. RT-3D, a much more user-friendly technique, now permits single-window and single-heart beat acquisition of complete LV segments in a volume-shaped cineloop [6], having the prerequisite to employed during stress echo. The aim of the present study was to assess the additional value of RT3D over conventional 2D dipyridamole stress echocardiography.

## Methods

### Patient population

The study population consisted of 23 consecutive patients (age = 65 ± 10 years, 16 men) with known or suspected coronary artery disease referred for clinically indicated stress echocardiography. Patients were prospectively enrolled in *Ospedale di Savona*, Italy with the following criteria: age ≥ 18 years; adequate echocardiogram to assess regional wall motion in 2D and RT-3D (the echocardiogram was considered adequate if ≥ 13 of the 17 segments were visualized). Exclusion criteria included: poor acoustic window, contraindications to dipyridamole, recent (< 1 month) episode of ventricular fibrillation, significant stenosis of the aortic valve, and patient refusal to enter the study. Decisions concerning medical therapy at the time of testing and/or coronary angiography were left to the attending physician. A quantitative luminal narrowing ≥ 50% was considered significant. All patients gave informed written consent prior to dipyridamole stress echocardiography.

### 2D imaging

Two-dimensional echocardiography images were performed with Sonos 7500 (Phillips Medical Systems, Palo, Alto, CA) equipped with a phased array 1.6–2.5 MHz probe with second harmonic capability. In all patients, the four standard views (parasternal long and short axis, apical 4 and 2 chambers) were obtained at baseline and peak stress and were recorded on super-VHS and digitally stored.

### Real-time 3D imaging

**Figure 1:**
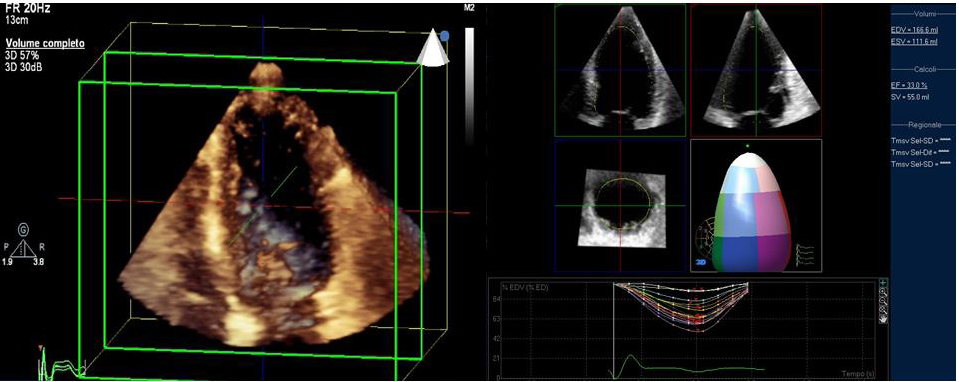
**Volumetric data obtained only from the apical RT-3D window and displayed as conventional 2D apical which where digitalized with final interpretation made off-line with the steering and tilting of the image planes for proper alignment and visualization of various scan**.

Real-time three-dimensional echocardiography images were recorded using Sonos 7500 (Phillips Medical Systems, Palo, Alto, CA) with 2–4 MHz matrix-phased array transducer in a 60 × 70 pyramid-shaped volume containing the entire LV. Volumetric data were obtained only from the apical window and displayed as conventional 2D apical which where digitalized with final interpretation made off-line with the steering and tilting of the image planes for proper alignment and visualization of various scan (Figure 1). Acquisition of baseline and peak 3D images were obtained after 2D studies using the same echocardiographic machine with a rapid switch (within 60 sec) between the 2 probes.

### Stress echocardiography

Patients were asked to abstain from food and drinks containing xanthine for ≥ 24 hrs prior to the study. All patients performed a dipyridamole stress echo. Dipyridamole was given at a maximal dose of 0.84 mg/kg in 10 minutes, unless symptoms of intolerability, positivity or hypotension (relative or absolute, >30 mmHg decrease in blood pressure) occurred. Test was considered positive for ischemia if any new or worsening dyssynergy occurred in more than 1 contiguous segment of the same vascular territory. A segment was considered to be viable when it improved by one grade or more at peak stress (for instance, a hypokinetic segment becoming normal, or an akinetic segment becoming hypokinetic). The left ventricle was divided into 17 segments as suggested the American heart Association [11]. Segmental wall motion was graded as follow: normal = 1, hypokinetic = 2, akinetic = 3, and dyskinetic = 4. Wall motion score index was derived by dividing the sum of individual visualized segment scores by the number of visualized segments. It has been agreed a priori to grade as normal any "mild" or "questionable" hypokinesia. Segmental image quality was assessed as previously reported [12] briefly a score of 1 = complete endocardial definition and wall thickening both at rest and at peak, 2 = visualization of all segments but not as well as [1], 3 = inadequate visualization ≤ 2 segments but adequate adjacent segments of same territory, 4 = inadequate visualization ≥ 3 segments but adequate adjacent segments of same territory, 5 = inadequate visualization comprising 1 or whole territories. Segmental analysis (wall motion and image quality) was done by joint reading of two expert observers on-line for 2D stress echo and off-line for RT-3D.

### Statistical analysis

Variables are expressed as mean values ± SD. Student paired-sample *t *test was used to compare continuous variables. Cohen's coefficient of variation, Kappa was used to assess agreement between 2D and RT-3D results. A Kappa value ≥ 0.45 was considered to be a good agreement while Kappa ≥ 0.75 was considered to be an excellent agreement. A p value < 0.05 was considered significant. All calculations were made using SPSS software (SPSS version 13.0 for windows, 1995).

## Results

**Figure 2:**
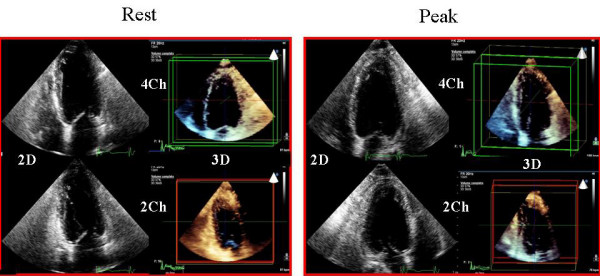
**Dipyridamole stress study showing 2-dimensional (2D) images and real-time 3-D images in rest condition (left side) and peak stress for the same patient**.

The clinical baseline characteristics, risk profile and medical therapy of the study population are outlined in Table 1. All patients tolerated the high dipyridamole dose (0.84 mg/kg), and no test was interrupted prematurely due to major side effects. One patient had diagnostic ST segment depression while 2 patients had typical angina during the test. Figure 2 represents an example of consecutive 2D and RT-3D imaging during dipyridamole stress echocardiography.

**Table 1:** Baseline characteristics

**Variables**	**n = 23 pts**
Age (yrs)	65 ± 10
Gender (M:F)	16:7
Hypertension	11 (48%)
Diabetes	4 (17%)
Hyperlipemia	8 (35%)
Smoking history	4 (17%)
	
**Previous CAD**	
MI	11 (48%)
PCI	2 (9%)
CABG	2 (9%)
	
*Angiographic results:*	
1-vessel disease	8
2-vessels disease	1
3-vessels disease	6
	
**Medical therapy**	
ACE inhibitors	10 (43%)
Beta-blockers	14 (61%)
Nitrates	8 (35%)
Calcium channel blockers	5 (22%)
Aspirin	20 (87%)
Anticoagulants	1 (4%)

### Feasibility of RT-3D echocardiography

**Figure 3:**
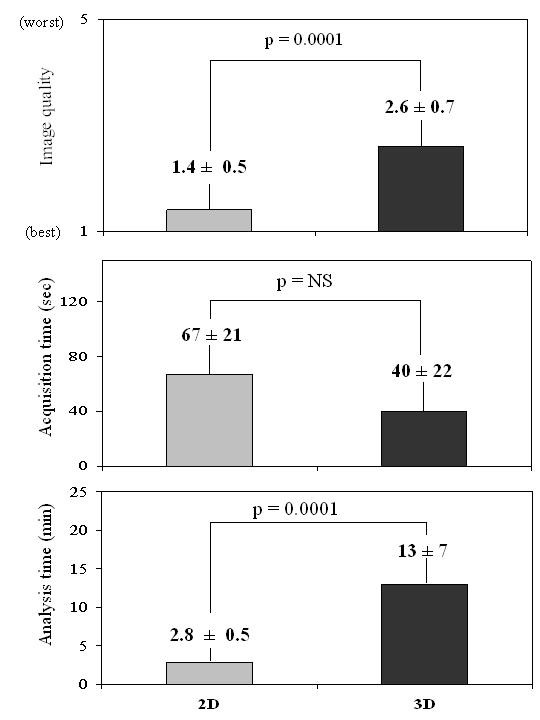
**Histograms showing the behavior of 2D and RT3D as for image quality, acquisition time, and analysis time**.

Image quality as defined by endocardium border detection was better with 2D than with RT-3D (Figure 3) leading to higher number of uninterpretable segments with RT-3D (18 segments for RT3D vs 7 segments for 2D). There was no statistical difference between acquisition time with either method but analysis time was shorter and on-line for 2D (Figure 3).

### Concordance 2D vs 3D: per patient analysis

**Figure 4:**
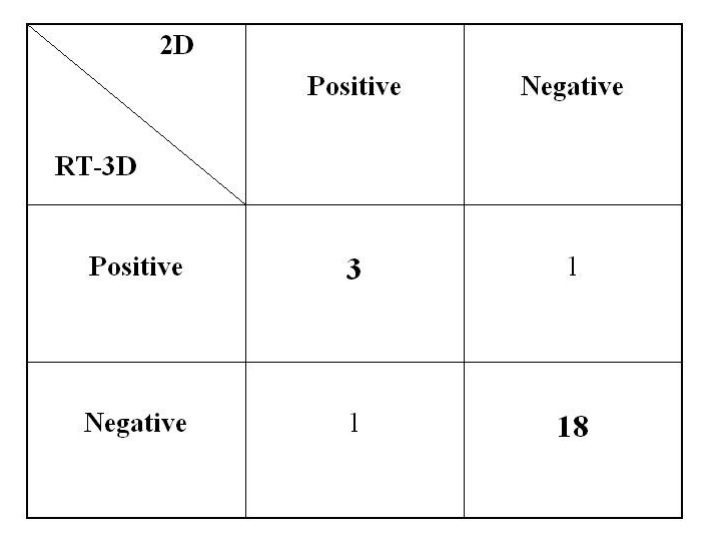
**2 × 2 table with the agreement between 2-dimensional (2D) and real-time 3-dimensional RT-3D) in the diagnosis of inducible ischemia**. Positive and negative results as assessed by 2D are displayed along the top, while RT-3D results are displayed along the left side. Complete agreement occurred in 21 patients.

**Figure 5:**
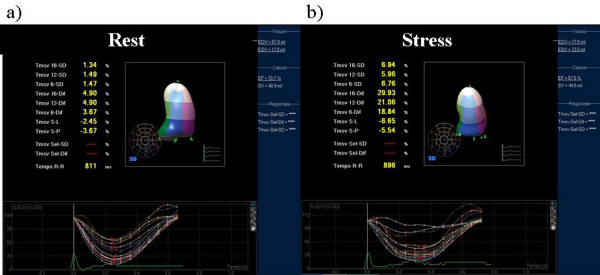
**Assessment of inducible ischemia with RT3D dipyridamole echo in a 65-years-old patients**. a) The still frame at baseline shows a normal systolic function (EF 60%). b) at peak stress dipyridamole 0,84 mg/Kg in 10 min the apical and median segments of the inferior wall and the median part of the inferior-lateral wall became akinetic with an abnormal displacement of the regional volumetric curves.

**Figure 6:**
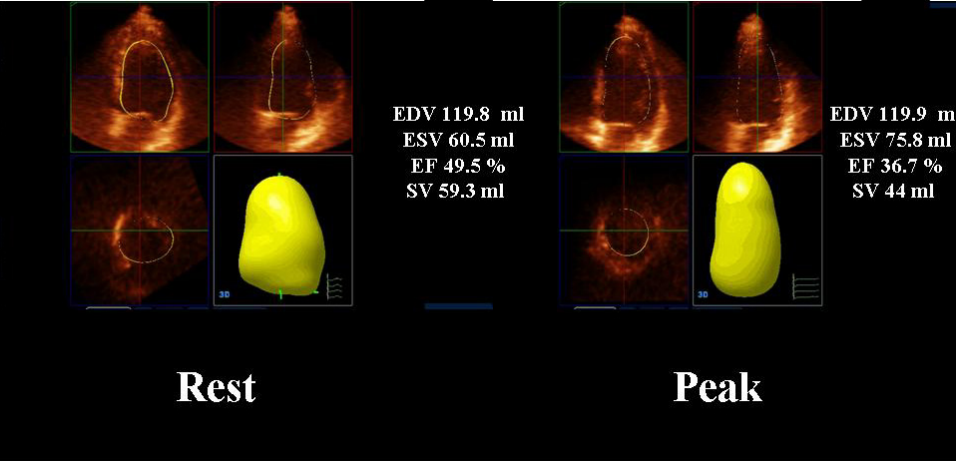
**Assessment of inducible ischemia with RT3D dipyridamole echo in a 64 years old man**. a) the movie at rest shows slightly decrease in ejection fraction at rest (EF 49.5%) without regional asynergy. b) The movie at peak stress the inferior wall and the inferior septum became akinetic with a decrease in EF (36.7%) due to an increase of left ventricular end systolic volume.

**Figure 7:**
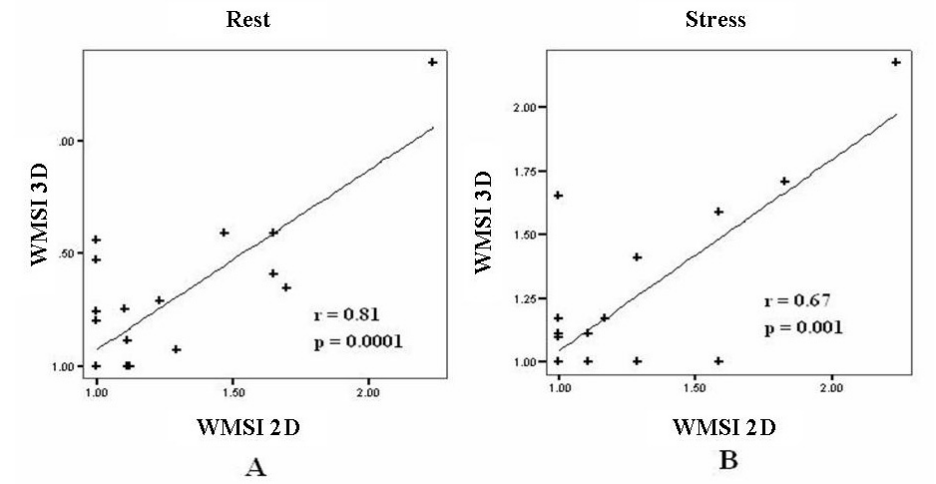
**Correlation between real-time 3-dimensional (3D) and 2-dimensional (2D) echocardiography as for assessment of wall motion score index (WMSI) for both a) rest and b) pea**k. An excellent linear correlation was obtained between the two techniques both at rest and at peak stress.

In total, 5 patients had a positive test for inducible ischemia in three of these five patients, diagnosis was made by both 2D and RT3D echocardiography, while one exam was positive only for 2D and one positive only for RT-3D (Figure 4, 5, 6; see additional file 1). Overall concordance (negative and positive results) was excellent between 2D and RT-3D for the diagnosis of ischemia with an agreement of 91% (kappa = 0.80). WMSI at rest was 1.20 ± 0.33 in 2D analysis and 1.23 ± 0.33 in RT-3D analysis, and WMSI at peak stress was 1.19 ± 0.32 for 2D and 1.23 ± 0.33 for RT-3D. A linear correlation was found between 2D and RT3D for wall motion analysis both at rest and at peak stress (Figure 7).

## Discussion

Real-time three-dimensional dipyridamole stress echocardiography is highly feasible in clinical practice. RT-3D allowed the rapid (~40 sec) acquisition at peak stress of a volumetric cineloop that contained the entire LV and to review multiple, standard 2D images. The inferior image quality of RT3D might partly be explained by the round and flat face of the transducer which offers poor intercostals skin contact and the low frequencies used [13]. Moreover the frame rates decrease further with an increase of heart rate (i.e.: during dobutamine infusion or during exercise) with consequent reduction of spatial resolution. In these study we used the dipyridamole 0.84 mg/kg in 10 minutes and the hemodynamic response to this drug is different in term of increase of heart frequency in comparison to dobutamine or exercise stress echo. For this reason the dypiridamole echo test could be the best choice in patients submitted to RT3D stress echo. In this study we showed a good to excellent concordance with 2D echo on wall motion analysis for detection of inducible ischemia.

## Comparison with previous studies

Previous studies on three-dimensional imaging used a different technology than real-time three-dimensional echocardiography, these previous techniques were dependant on both ECG and respiratory gating because 3D images were a composite of multiples 2D acquisitions and cardiac cycles, thus highly affected by translation motion, arrhythmia and improper spatial registration [6]. Due to these limitations they never entered the clinical practice.

Real-time three-dimensional echocardiography has the prerequisites to analyze segmental wall motion, volume, etc. In fact, it has been demonstrated that [6,13-18] using real-time technology it was possible to image, more rapidly, most of LV segments by a single-window acquisition: 93–98% of LV segments from parasternal volume set and 85–89% from apical volume set [6,14]. It was also demonstrated that RT-3D had a high concordance (Kappa = 0.52–0.72) with 2D reading [14]. In our study, we achieved similar rates of LV segments visualization but there was only a non significant trend toward a shorter time of acquisition with RT-3D when compared with 2D which might be accounted for an appropriate learning curve time. The time necessary to RT3D imaging optimization was measured and it may have prolonged significantly the acquisition time.

RT3D stress echocardiography has been previously shown to be highly feasible for both dobutamine [14-18] and exercise [19] stress testing but the true incremental value was never proven and only one study showed higher sensitivity than 2D imaging (87.9% vs 79.3%) [14]. In the present we did not address the diagnostic accuracy of RT-3D vs 2D echo but other authors have demonstrated comparable sensitivity (93%), specificity (75%), and accuracy (99%) between the two methods [16].

**Figure 8:**
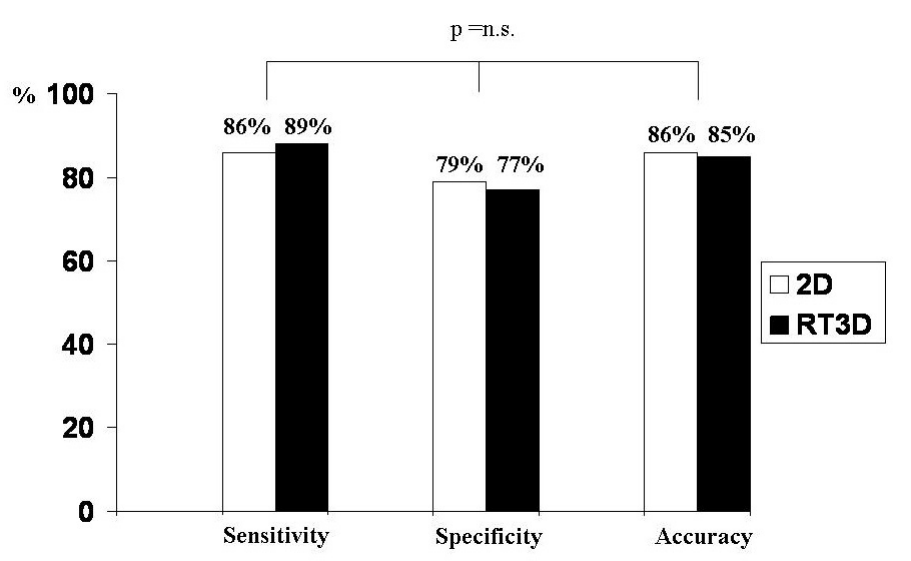
Sensitivity, specificity and accuracy of 2D (white bar) and 3D (black bar) dobutamine stress echocardiography (from 14–17).

Inferior spatial resolution and frame rates compared with 2D echocardiogram are the disadvantages of RT3D echo. These drawbacks may offset the advantages of RT3D echo (offline manipulation of real three dimensional images; ability to obtain multiple sections of any desired segments; virtual elimination of off-axis acquisition; shorter acquisition time, and so on) and could explain why RT3D has not been shown to be superior to 2D echo in previous reports (Figure 8).

## Clinical Implications

RT3D has entered the clinical arena but no additional value over conventional 2D echocardiography during stress echocardiography can be demonstrated. Recent ASE and EAE consensus statements on stress echocardiography did not recommend the routine use of this technology during stress echocardiography even though it may shorten significantly time of acquisition counterbalanced by a longer time of data-set analysis. Nonetheless RT3D may play a role in stress echocardiography when wall motion analysis is not the target. The assessment of contractility or the function of the right ventricle can become the perfect clinical setting for this technique. More studies are warranted to assess its real clinical value in different subsets of patients but the evidence is sound enough to state that RT3D is here to stay.

## Authors' contributions

SV acquired most images, LP and PS analyzed 3d data, AG and MB contributed to data collection, PB critically revised the manuscript. All Authors have read and approved the final manuscript.

## Supplementary Material

Additional file 1 AVI 1.avi. stress echo (basal, peak). Click here for file
